# Exploring NMR ensembles of calcium binding proteins: Perspectives to design inhibitors of protein-protein interactions

**DOI:** 10.1186/1472-6807-11-24

**Published:** 2011-05-12

**Authors:** Adriana Isvoran, Anne Badel, Constantin T Craescu, Simona Miron, Maria A Miteva

**Affiliations:** 1 MTi, Inserm U973 - University Paris Diderot, 35 rue Helene Brion, Bat. Lamarck, 75013 Paris, France; 2 Department of Chemistry, West University of Timisoara, 16 Pestalozzi, 300115 Timisoara, Romania; 3 Institut Curie Centre de Recherche, Centre Universitaire Paris-Sud, 91405 Orsay cedex, France; 4 Inserm U759, Centre Universitaire Paris-Sud, 91405 Orsay cedex, France

## Abstract

**Background:**

Disrupting protein-protein interactions by small organic molecules is nowadays a promising strategy employed to block protein targets involved in different pathologies. However, structural changes occurring at the binding interfaces make difficult drug discovery processes using structure-based drug design/virtual screening approaches. Here we focused on two homologous calcium binding proteins, calmodulin and human centrin 2, involved in different cellular functions via protein-protein interactions, and known to undergo important conformational changes upon ligand binding.

**Results:**

In order to find suitable protein conformations of calmodulin and centrin for further structure-based drug design/virtual screening, we performed *in silico *structural/energetic analysis and molecular docking of terphenyl (a mimicking alpha-helical molecule known to inhibit protein-protein interactions of calmodulin) into X-ray and NMR ensembles of calmodulin and centrin. We employed several scoring methods in order to find the best protein conformations. Our results show that docking on NMR structures of calmodulin and centrin can be very helpful to take into account conformational changes occurring at protein-protein interfaces.

**Conclusions:**

NMR structures of protein-protein complexes nowadays available could efficiently be exploited for further structure-based drug design/virtual screening processes employed to design small molecule inhibitors of protein-protein interactions.

## Background

Protein-protein interactions (PPIs) are important for regulating many biological functions. It has been suggested that the human interactome involves about 650,000 interactions [1] and disrupting these interactions could be an attractive way to block a number of targets involved in different pathologies [2,3]. A possible strategy to inhibit undesired PPIs is to design small organic molecules binding in the zone of interactions and the increasing number of such recent success stories prove it [3-5]. Yet, it is difficult to efficiently target PPIs due to large and flat interfaces [6], the nature of the chemicals present in chemical libraries [7,8], and in particular due to the structural changes that can occur upon ligand binding. In some cases, small structural changes have been observed at the PPIs interfaces due to small inhibitors' binding [5]. Other proteins, i.e. calmodulin, undergo considerable conformational changes due to protein or small ligand binding [9]. Indeed, limitations in describing potential small-molecule binding sites have been noted when using static structures of either the unbound protein or the protein-protein complex [6].

Some early designed inhibitors of PPIs mimic short secondary-structural elements of proteins [2]. Other molecules, like the terphenyl and its derivates (mimicking alpha-helical regions), were shown to be able to inhibit several PPIs [10,11], e.g. terphenyls disrupt the calmodulin (CaM) interactions with smooth muscle myosin light-chain kinase (smMLCK), with 3'-5'-cyclic nucleotide phosphodiesterase, or with the helical peptide C20W of the plasma membrane calcium pump [12].

**Figure 1:**
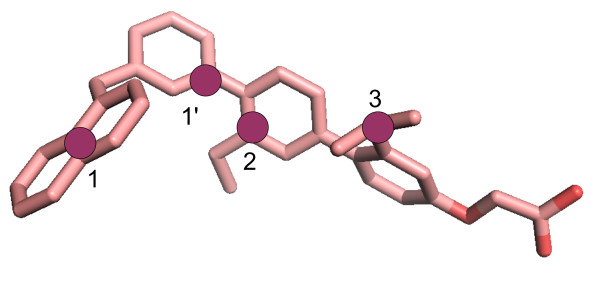
**1-naphthyl therphenyl structure colored by atom type**. The pharmacophoric points chosen for docking accuracy evaluation are shown as purple circles for CaM: 1, 1', and 2, and for HsCen2: 1, 2, and 3.

**Figure 2:**
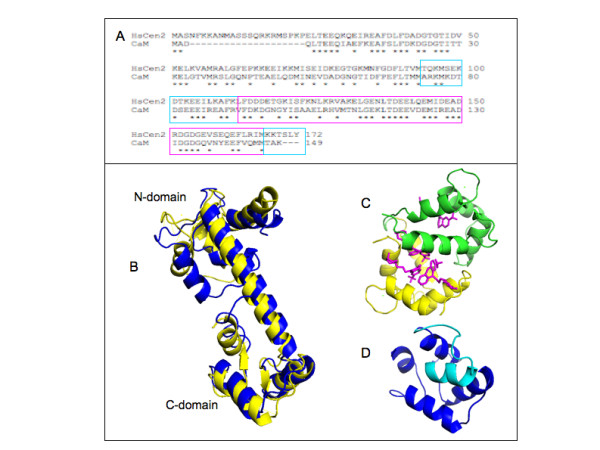
**Sequence and structural homology of calmodulin and centrin**. (A) sequence alignment of CaM and HsCen2, the C-terminal domains are shown in blue and the pocket regions in magenta; (B) superposition of the X-ray structures of CaM (yellow cartoon, unbound form, code 1CLL) and HsCen2 (blue cartoon, bound form, code 2GGM); (C) CaM in a complex with trifluorperasine (sticks in magenta) (code 1LIN); (D) structure of HsCen2 (unbound form, code 1M39). The blue region corresponds to the C-domain of HsCen2; the helix (in cyan) belongs to the N-terminal domain.

We exploit here docking of 1-naphthyl terphenyl (see Figure 1) into two homologous Ca^2+^-binding proteins, CaM and human centrin 2 (HsCen2), to find out the CaM and HsCen2 conformations that could efficiently be employed for further structure-based design of inhibitors of PPIs. CaM and HsCen2 have a high sequence homology (Figure 2A) and display a structural similarity as both proteins are composed by two EF-hand N- and C-terminal domains connected by a helical linker (see Figure 2B). The binding of 1-naphthyl terphenyl by CaM (IC50 = 9 nM) has already been shown experimentally [12]. Following the strong similarity between the two Ca^2+^-binding proteins we probe in this study a potential terphenyl binding into HsCen2.

CaM is expressed in all eukaryotic cells and interacts with a large number of different protein targets [13], being thus involved in regulation of different cellular processes, such as cell division and differentiation, ion transport, muscle contraction, etc. [14,15]. Ca^2+^-binding induces a rearrangement of the tertiary structure of EF-hand domains of CaM [16] with an exposure of a large hydrophobic cavity promoting the association of a wide array of target proteins, including kinases, cyclases, various cell surface receptors, etc. CaM displays a multitude of conformational states [17-19]. Modulation of physiological targets of CaM through CaM inhibition by small natural or synthetic compounds [20] may guide discovery of new therapeutic agents.

Centrins are involved in the centrosome duplication [21], in the nuclear excision repair (NER) mechanism [22] or in the multiple nuclear export pathways [23]. NER is an essential molecular mechanism responsible for repairing of DNA lesions caused by UV light or antitumor agents like cis-platin. Cis-platin resistance in chemotherapy is a major complication in cancer and seems to be associated with the stimulation of NER DNA repair mechanism [24]. Centrin forms a heterotrimeric complex with XPC (*Xeroderma pigmentosum *group C) and hHR23B proteins, which play a key role in the DNA damage recognition. Recent *in vivo *and *in vitro *studies [25,26] revealed that HsCen2 binds to a 17-mer peptide (N847-R863) of XPC protein (P17-XPC) with a high affinity in the presence of Ca^2+ ^(~ 10^8 ^M^-1^). Human cell lines expressing a mutant XPC protein (in the centrin binding motif) exhibited *in vitro *and *in vivo *a significant reduction of NER activity [25]. Thus, inhibition of centrin-XPC interactions involved in the NER mechanism might be an efficient way to modulate these processes.

Structural changes occurring at PPIs interfaces make difficult to successfully proceed to structure-based drug design/virtual screening of novel small molecules inhibiting PPIs [27-29]. Selecting appropriate conformations, taking into account the protein plasticity, could be a valuable starting point for subsequent structure-based virtual screening studies. One possibility to incorporate the protein receptor flexibility for ligand docking is to explore multiple receptor conformations (MRC) [30,31], either experimental [32-34] or modeled [35-39]. Once the MRC selected, ligand candidates can be docked into each receptor conformation and the results from each docking run can be combined together in a post-processing step [40]. Recent papers showed examples of using NMR ensembles of the protein receptor for docking and screening processes [33,41]. In this work we performed *in silico *analysis and docking of 1-naphthyl terphenyl into NMR ensembles of CaM and HsCen2 that revealed a small set of NMR conformations appropriate to perform further structure-based virtual screening for discovering of small PPIs inhibitors.

## Results and Discussion

### Protein-protein binding site analysis

CaM and HsCen2 share about 50% sequence homology extending even to the positions of side chains in the hydrophobic core of the proteins. The main difference between them is the presence in HsCen2 of a 25 amino acids N-terminal ending region (Figure 2A). Both proteins possess four EF-hands, but for HsCen2 only the EF hands belonging to the C-terminal domain bind Ca^2+ ^ions [42] with a significant affinity (~ 10^4^-10^5 ^M^-1^). We should note the high sequence homology of the C-terminal domains of these two Ca^2+^-binding proteins (blue and purple regions in Figure 2A), especially in the binding sites (purple regions in Figure 2A). The superposition of CaM and HsCen2 structures shows their strong structural similarity (Figure 2B). The root mean square deviations (RMSD) between the carbon alpha atoms of the CaM and HsCen2 structures shown in Figure 2B is 3.3 Å, whereas the RMSD between the two C-terminal domains is 0.8 Å.

The flexible helical linker between N- and C-terminal domains enables the switch between different conformational states of CaM and HsCen2. Figures 2B and 2C show two conformations of CaM, namely an "extended" mode (in the absence of ligand) and a "wrap-around" mode (in a complex with trifluorperasine), respectively. In the last one, the central helix becomes partially unstructured and the helices of the N-terminal domain point toward the bound trifluorperasine molecules. It has been demonstrated that the C-terminal domain of CaM (C-CaM) binds several peptides/proteins [43]. Similarly, the terphenyl molecule (mimicking the CaM-binding face of smMLCK), binds exclusively into the C-domain of CaM [12]. The residues W4, T7 and V11 of smMLCK (noted as i, i + 3, i + 7 of the alpha-helix) are critical for the interaction with C-CaM [16].

Similarly, HsCen2 undergoes important conformational changes depending on the presence of a bound ligand (see Figure 2B and 2D) [18]. In the HsCen2/P17-XPC complex, the alpha-helical linker between the two domains undertakes an extended form (Figure 2B), and in the unliganded form the same region closes the C-terminal peptide binding site (Figure 2D). Structural studies showed that HsCen2 binds the 17-mer XPC peptide only by its C-terminal domain and the W2, L5 and L9 residues (1-4-8 motif) of the P17-XPC have been shown as critical anchoring side chains [26,44]. Thermodynamic studies [26] enabled the definition of a minimal centrin binding site, a peptide of five residues, which accounted for about 75% of the total free energy of interaction between the two proteins.

**Figure 3:**
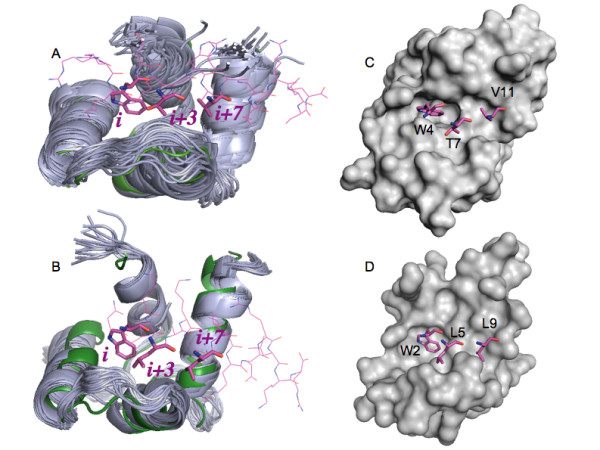
**Superposition of the C-terminal domains of CaM and HsCen2 used for docking**. (A) 31 NMR (code 2K0F; in grey cartoon) and one X-ray (code 1CLL; in green cartoon) structures of CaM. One of the NMR smMLCK peptide structures bound to CaM is shown in pink (code 2K0F); (B) 20 NMR (code 2A4J; in grey cartoon) and one X-ray (code 2GGM; in green cartoon) structures of HsCen2. The peptide P17-XPC bound to HsCen2 is shown in pink (code 2GGM); (C) surface presentation of the X-ray structure of CaM (code 1CLL); (D) surface presentation of the X-ray structure of HsCen2 (code 2GGM). The sticks in pink represent the positions of the peptide residues *i, i + 3, i + 7 *key for the binding.

The above presented data indicate that the C-terminal domains of both Ca^2+^-binding proteins are more functional regarding the peptides binding. Therefore, we explored the C-terminal domains of CaM and HsCen2 for potential small ligands' binding. We analyzed several X-ray structures and NMR ensembles of both proteins to construct a relevant ensemble of multiple receptor conformations for the docking process of 1-naphthyl terphenyl. The selected sets contained crystal structures as well as 31 NMR structures (among 160 ones) and 20 NMR structures for C-CaM and C-HsCen2, respectively (see for details the Methods section). The selected NMR and X-ray structures of C-CaM and C-HsCen2 are shown in Figure 3. The residue numbers correspond to the ones in the NMR files, 2K0F for CaM and 2A4J for HsCen2.

### Docking of terphenyl

**Figure 4:**
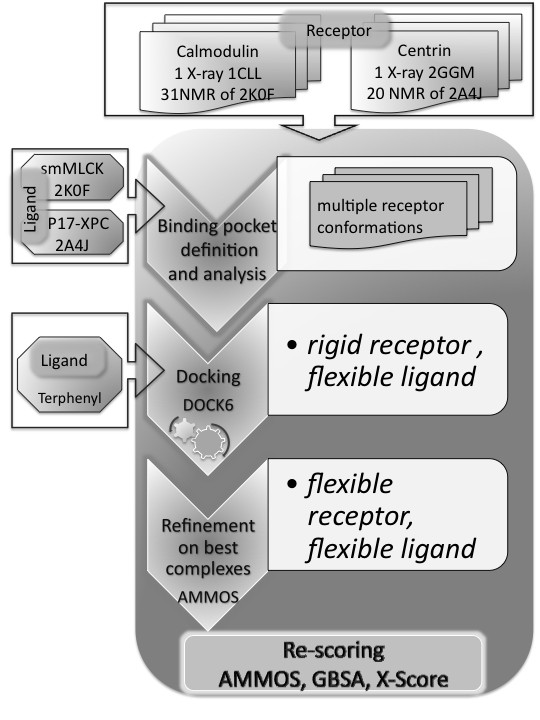
**Flowchart of the employed docking-scoring protocol**.

**Figure 5:**
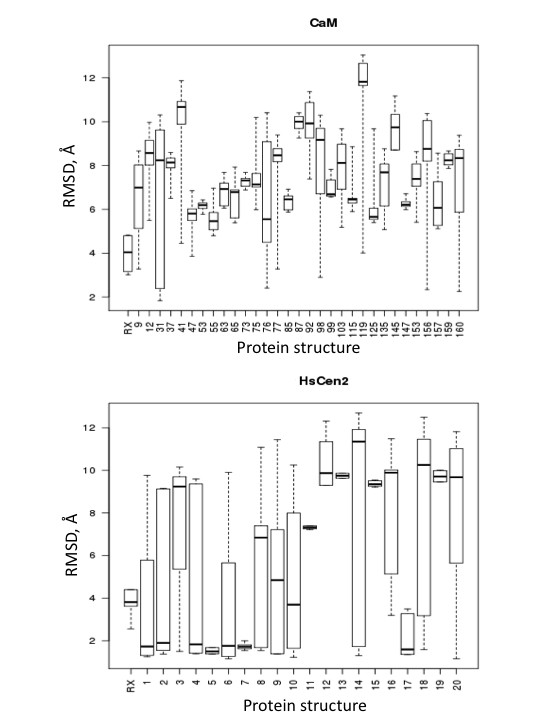
**Box plots of RMDS values (Å) between the docking poses of terphenyl and the reference pharmacophoric points**. The box region corresponds to the 50% of the distribution, with shown median. (A) docking into the X-ray (code 1CLL) and the 31 NMR structures (code 2K0F) of CaM; (B) docking on the X-ray (code 2GGM) and the 20 NMR structures (code 2A4J ) of HsCen2.

The docking-scoring protocol employed to dock 1-naphthyl terphenyl into the selected structures is shown in Figure 4 (see for details in the Methods section). In order to identify the "best" protein conformations for further analysis, we calculated the RMSD between each pose obtained after docking with DOCK6.0 [45] and the reference points of smMLCK and P17-XPC for CaM and HsCen2, respectively (see Figure 1). The obtained RMSD values are shown in Figure 5A and 5B. Overall, docking results are best for the structures of C-HsCen2. We compared the binding zones of the two proteins to analyze these results. For C-CaM, the binding pocket consists of one cavity (volume 314.22 Å^3^, code 1CLL) containing residues F88, I96, L101, M105, M120, E123, M140 and M141. The residue F88 placed in the center of the binding zone is in contact with W4 and T7 of the smMLCK peptide. The binding site of HsCen2 is larger (volume 417.27 Å^3^, code 2GGM) and consists of two hydrophobic cavities separated by F113 interacting with L5 of the P17-XPC peptide, and L126 and M145 interacting with W2 of the peptide. The close contact of F113 and L5 of the bound peptide has also been observed in the structure of HsCen2 complexed with another protein partner targeting the same HsCen2 zone [46]. The deeper and bigger cavity contains the residues F113, I146, E148, V157, I165 and M166, and the smaller one contains the residues L126, V129, A130, L137, L142 and M145. The replacement of one Met residue (M105) of C-CaM with a smaller one, an Ala residue (A130), enlarges the hydrophobic cavity of the C-HsCen2. This facilitates a potential anchoring of 1-naphthyl terphenyl into the C-HsCen2.

We also compared the flexibility of the binding zone of CaM and HsCen2, by analyzing the B-factors of the carbon alpha atoms for all residues in the binding pocket of HsCen2 complexed with P17-XPC, as well as for a few complexes of human CaM interacting with helical peptides of similar length as P17-XPC (PDB codes: 3EWV, 1IWQ, 2VAY, 1ZUZ, 3BYA, and 1YR5). This analysis showed an enhanced flexibility of CaM in a bound state, in the region 107-113 compared to the binding zone 132-138 of HsCen2. Structural comparison of these complexes suggested that this difference would be mainly due to a higher mobility of the K111 side chain of CaM compared to N136 of HsCen2. Moreover, we should note the presence of four Met residues in the binding site of C-CaM (M105, M120, M140, M141) and two Met residues in the pocket of C-HsCen2 (M145 and M166). The flexible nature of the Met side chains at the binding surface has previously been discussed as a critical factor to facilitate the surface complementarity between CaM and its partner [19,47]. This analysis shows a higher plasticity of the binding pocket of the C-CaM than the C-HsCen2, and, therefore, more structural arrangements might occur for the C-CaM than for the C-HsCen2 upon ligand binding.

**Figure 6:**
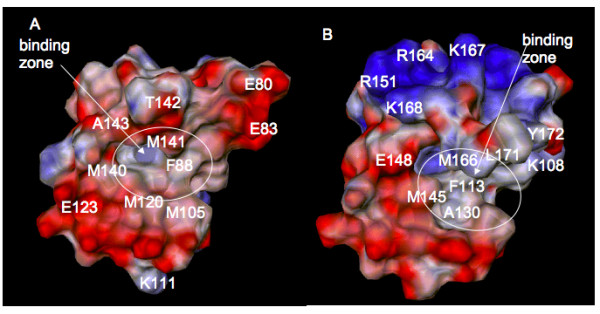
**Electrostatic potential distribution on the protein surface of the C-terminal domains**. (A) CaM; (B) HsCen2. The potential computed using PCE [59] is shown from -3.0 kcal/mol/e (red) to +3.0 kcal/mol/e (blue). The residue numbers correspond to the ones in the NMR files, 2K0F for CaM and 2A4J for HsCen2.

The 3D electrostatic potential distribution on the X-ray C-CaM and C-HsCen2 surfaces (see Figure 6) indicates that overall C-CaM is more negatively charged than C-HsCen2; this could be related with the stronger affinity of Ca^2+ ^for CaM than HsCen2 [48]. This observation is also valid for the binding zone of the C-CaM and C-HsCen2. The presence of a large number of negatively charged residues in both proteins, and especially in C-CaM, resulted in several computed abnormal pKa values for C-CaM: 7.3 for E100, 8.4 for D129, and 7.6 for E136; for HsCen2: 6.6 for D114 and 7.4 for D154 (these residues are not situated in the binding pocket). The mean local hydrophobic density calculated using Fpocket tool [49] was 41.39 and 54.78 for the binding pockets of C-CaM and C-HsCen2, respectively. Following these results, we can speculate that the higher hydrophobicity of C-HsCen2 binding zone might facilitate a potential binding of the hydrophobic 1-naphthyl terphenyl.

The RMSD results allowed to retain for further analysis five best NMR models for the C-HsCen2 and C-CaM, in addition to the X-ray structures (for CaM:2K0F: models 31, 76, 98, 156 and 160; for HsCen2:2A4J models 1, 5, 6, 7 and 17). As can be seen for both proteins better docking poses were obtained when docking on some of the NMR conformations compared to the X-ray ones. The binding pockets of the five best NMR models have larger volumes than the X-ray structures for both proteins. For C-CaM, the cavity is deeper in the selected NMR models (e.g. the volume is 399 Å^3 ^for the model 31) than in the X-ray structure (volume 314 Å^3^). The binding pocket of the X-ray structure of HsCen2 (volume 417 Å^3^) is much smaller than those of the best five NMR models (volumes: 1043 Å^3 ^for model 1, 934 Å^3 ^for model 5, 1419 Å^3 ^for models 6, 1277 Å^3 ^for models 7 and 1134 Å^3 ^for model 17), that obviously makes easier the terphenyl docking into these NMR structures. We suggest that this observation would be valid as well for other small ligands' docking. The large difference between the pocket volumes of the best NMR models and X-ray structure of C-HsCen2 is due to the orientation of two residues, F113 and F162, that fill a large part of the binding cavity in the X-ray structure. Similar situation was observed for C-CaM and F88.

### Poses' refinement and interaction energy analysis

**Figure 7:**
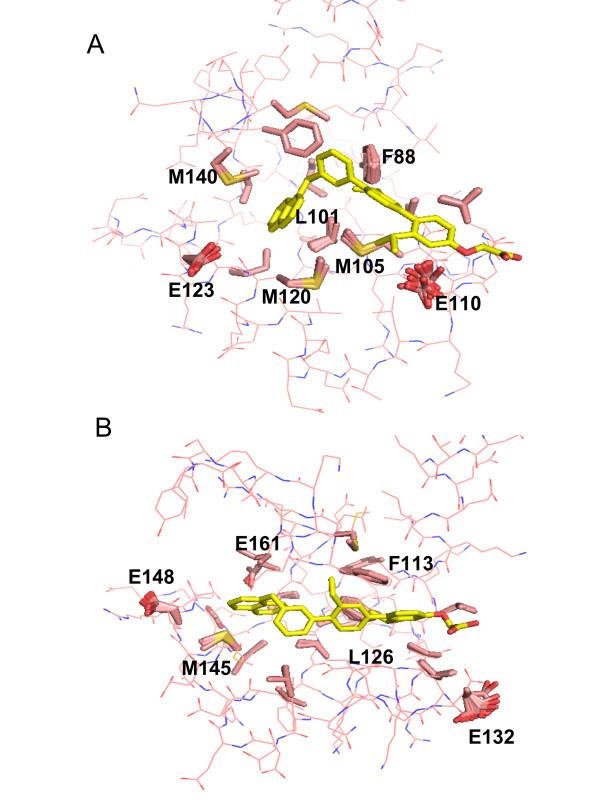
**Side chains of the protein residues moving after the refinement with AMMOS (in pink sticks)**. The top AMMOS scored poses of terphenyl are shown in yellow sticks. (A) CaM, the 2K0F model 31; (B) HsCen2, the 2A4J model 5.

As previously shown [50,51], post-docking optimization may help to further improve both docking poses and scores. We performed additional energetic analysis (see Tables 1 and 2) of docking poses on the selected best MRC to optimize the predicted binding modes and to re-calculate the interaction energies taking into account desolvation effects due to ligand binding. Firstly, we carried out an energy minimization of the docking poses on the selected NMR conformations and X-ray structures of both proteins using the program AMMOS [52]. The included flexible side chains of the protein receptor around the bound terphenyl enabled to relax the complex structures in the binding pocket. The energy gain due to the AMMOS relaxation for the best scored poses is shown in Tables 1 and 2. The important energy decrease during this step is due to reducing clashes between the docked ligand and some residues of the protein binding pocket, as well as internal ligand energy optimization. Figure 7 represents the side-chain orientations after the energy minimization for the different docking poses. The residues slightly moving due to the optimization are F88, L101, M105, E110, M120, E123, and M140 for CaM (Figure 7A) and F113, L126, E132, M145, E148 and E161 for HsCen2 (Figure 7B). Interestingly, it can be seen that Met residues M105, M120 and M140 are among the moving residues, as discussed above. As seen in Figure 7, the changes due to the optimization are not very large, still small variations of the docked complex structure can affect the interaction energy prediction (see Tables 1 and 2, the AMMOS energy, before and after AMMOS refinement). It has been previously discussed that even small receptor movements can lead to important modifications into the molecular recognition pattern and/or binding energy prediction errors [53]. To this end, the AMMOS refinement step could be useful to "rescue" some docking poses with bad energy score after docking on a rigid receptor (e.g. the docking pose 19 on the 2K0F model 160, Table 1).

**Table 1:** Terphenyl-CaM interaction energy (in kcal/mol) predicted by the methods DOCK, AMMOS, GBSA, X-Score for the top scored poses

	RMSD (Å)	DOCK vdw + es	AMMOS vdw + es		GBSA vdw + es	X-Score
					
Receptor structure No pose			before AMMOS refinement	after AMMOS refinement		
1CLL						
1	3.3	-48.63	-14.90	-26.09	-52.53	5.95
2	4.8	-48.27	-9.71	-26.72	**-61.12**	6.31
3	3.0	-48.15	-18.32	**-29.91**	-56.85	**6.38**

2K0F model 31						
1	1.9	-42.70	-13.04	-**36.42**	-40.05	**6.78**
2	8.8	-41.70	-12.64	-26.45	**-46.17**	6.68

2K0F model 76						
1	4.5	-48.12	-4.24	-22.43	-31.43	6.24
5	3.3	-45.71	-20.72	**-34.51**	-46.11	**6.86**
8	3.1	-44.68	-20.23	-30.63	**-49.13**	6.81

2K0F model 98						
1	8.8	-49.79	3.37	-16.05	-21.64	6.14
16	7.9	-38.89	5.71	**-17.65**	-23.62	5.77
17	7.4	-38.32	48.93	-4.83	**-31.53**	**6.34**

**2K0F model 156**						
1	8.2	-50.52	-6.90	-13.17	-34.42	4.31
6	2.3	-45.57	-13.08	**-36.31**	-48.90	4.69
12	2.5	-43.51	-19.25	-34.35	**-49.93**	**4.89**

2K0F model 160						
1	8.7	-41.13	3.15	-4.23	-40.65	4.81
2	5.3	-40.57	1.36	-6.20	**-49.48**	4.64
19	2.3	-19.66	43.66	**-41.25**	-38.44	**5.03**

The column 2 shows the RMSD of the poses as predicted by DOCK.

**Table 2:** Terphenyl-HsCen2 interaction energy (in kcal/mol) predicted by the methods DOCK, AMMOS, GBSA, X-Score for the top scored poses

	RMSD (Å)	DOCK vdw + es	AMMOS energy vdw + es		GBSA vdw + es	X-Score
					
Receptor structure No pose			before AMMOS refinement	after AMMOS refinement		
2GGM						
1	2.7	-56.10	>2000	**-38.74**	-72.47	**7.01**
13	2.2	-49.02	>2000	-19.93	**-74.13**	6.56

2A4J model 1						
1	1.3	-69.63	-33.45	**-56.14**	-66.68	8.19
2	1.4	-67.60	-30.10	-50.40	-67.88	**8.21**
7	9.7	-63.23	-34.75	-39.31	**-76.58**	7.66

**2A4J model 5**						
1	1.8	-65.07	-20.30	-46.75	**-67.91**	7.80
4	1.6	-63.82	-18.92	**-48.72**	-59.92	**8.26**

**2A4J model 6**						
1	1.7	-64.29	29.11	**-49.35**	-69.97	7.99
3	1.9	-62.29	-15.63	-39.59	-67.46	**8.00**
9	1.8	-60.66	37.90	-47.70	**-70.74**	7.98

**2A4J model 7**						
1	1.6	-65.88	-23.25	**-41.74**	-73.64	8.16
2	1.6	-65.09	-19.22	-38.49	**-75.20**	**8.31**

**2A4J model 17**						
1	1.5	-66.54	145.48	-28.03	-**79.76**	8.08
7	1.7	-63.64	635.45	**-41.46**	-76.38	7.79
10	1.4	-63.18	>2000	-35.88	-77.15	**8.21**

The column 2 shows the RMSD of the poses as predicted by DOCK.

**Figure 8:**
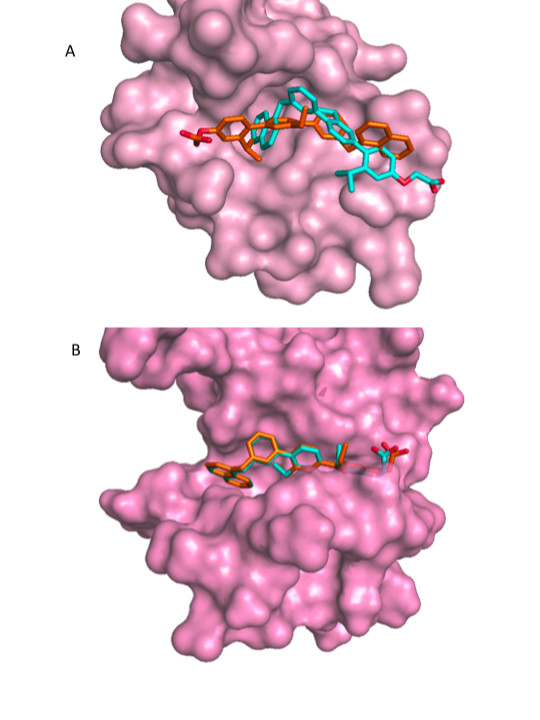
**Superposition of the top scored poses of terphenyl**. The poses after docking-scoring with DOCK6.0 are shown in cyan and the poses after the GBSA re-scoring are shown in orange. (A) CaM, the 2K0F model 31; (B) HsCen2, the 2A4J model 5.

Further, we employed re-scoring with the GBSA Hawking model as implemented in DOCK6.0, and re-scoring with X-Score [54] on the AMMOS optimized docked complex structures. Tables 1 and 2 show the top scored poses retrieved by each of the methods: DOCK, AMMOS, GBSA and X-Score. We consider docking poses of bound terphenyl within 2.5 Å RMSD as acceptable. The best protein conformations for CaM and HsCen2 were found by consensus between AMMOS, GBSA and X-Score re-scoring if the best score corresponds to a good RMSD pose. Among the twelve protein structures, 1CLL, 2K0F model 76, and 2K0F model 98 were considered as "bad" because no one of the re-scoring methods AMMOS, GBSA and X-score retrieved good docking poses. The AMMOS energy and X-Score retrieved good docking poses for 8 out of the 9 remaining acceptable cases. GBSA scoring found good poses for 6 out of the 9 acceptable cases. Figure 8 illustrates one good and one bad solutions found by GBSA. In the case of NMR model 5 of C-HsCen2, (Figure 8B), GBSA retrieved a good docking pose. However, for the NMR model 31 of C-CaM, (Figure 8A), the pose retrieved by GBSA is completely upside-down. GBSA failed to find the good poses in 3 out of 9 possible cases. Similar problems (taking into account more complex physical terms, *via *PBSA or GBSA methods, that demand precise positions of the protein and ligand atoms) have already been found in other docking studies [51,55]. It might be possible that a further optimization of the docked complexes including an implicit solvent or explicit water molecules during the minimization would be useful for a more successful re-scoring with the GBSA method.

The results in Table 1 reveal the best C-CaM conformations suitable for further structure-based drug design/virtual screening: the best one is 2K0F model 156 where the good docking poses were found by the three re-scoring scoring methods; the models 2K0F 31 and 160 are acceptable with good poses found by AMMOS and X-Score. In the case of HsCen2 (Table 2), the 2A4J models 5, 6, 7, and 17 appear to be best ones where the three re-scoring methods retrieved the good docking poses; the 2A4J model 1 is acceptable with good poses found again by AMMOS and X-Score.

## Conclusions

This work highlights that scoring and docking accuracy strongly depend on considering the receptor flexibility, either large conformational changes or small side-chain adjustments in the protein-protein binding region occur. Exploiting the NMR ensembles could be very helpful to take into account the receptor conformational changes into docking/virtual screening exercises. Local induced-fit optimization in a protein-ligand complex structure can be achieved by using the AMMOS method. We explored docking of terphenyl on a number of NMR conformations *vs *X-ray structures of CaM and HsCen2. Using the NMR ensembles of the receptor structure substantially improved the docking and scoring compared to the X-ray structures. Our study provided a minimal set of conformations of CaM and HsCen2 suitable for small ligand docking/virtual screening targeting the CaM and HsCen2 interactions. The comparative structural and energetic analysis of the binding sites of both proteins demonstrate large similarities and some differences. All together these data can be valuable for a future design of small PPIs inhibitors for CaM and HsCen2.

## Methods

### Selection of CaM and HsCen2 structures and binding pocket analysis

X-ray structures and NMR ensembles of CaM and HsCen2, all in the Ca^2+^-bound state, have been taken from the Protein Data Bank [56] and analyzed in details as follows: i) For CaM: an unliganded X-ray structure, code 1CLL at 1.7 Å resolution [57]; a NMR ensemble of 160 unliganded structures, code 2K0E [58]; a NMR ensemble of 160 structures bound to 19-mer peptide from smMLCK, code 2K0F [58]; ii) For HsCen2: a NMR ensemble of unliganded C-terminal domain, code 1M39; a X-ray structure of HsCen2 bound to the P17-XPC peptide, code 2GGM at 2.35 Å resolution [44]; a NMR ensemble of 20 structures of HsCen2 bound to P17-XPC, code 2A4J [48].

For CaM, the X-ray structure of the human unliganded CaM (1CLL) with the highest resolution among other retrieved X-ray CaM structures (PDB codes: 1LIN, 3EWV, 1IWQ, 2VAY, 1ZUZ, 3BYA, 1YR5) has been considered for docking calculations. We selected the NMR ensemble 2K0F for docking experiments as the key for the binding residues i, i + 3, i + 7 (W4, T7 and V11) (see Figure 3) [16] of the bound helical peptide smMLCK can be mimicked by the docked 1-naphthyl terphenyl.

For HsCen2, we have taken the X-ray structure of HsCen2 extracted from the complex with the P17-XPC peptide (code PDB 2GGM). In the NMR ensemble 1M39, the helix F86-Q95 enters in the binding site and closes the conformation. For 2A4J, the C-terminal domain of HsCen2 is in an open conformation and the binding site is occupied by the side chains of the bulky hydrophobic residues W2, L5 and L9 of P17-XPC. Taking into account that 1-naphthyl terphenyl mimics the binding motif i, i + 3, i + 7 (residues W2, L5 and L9) of P17-XPC, we have considered the 2A4J ensemble for our docking experiments.

The superposition and the analysis of all mentioned structures when focusing on the protein binding sites of CaM and HsCen2, revealed that the pockets are quite similar in the NMR ensembles 2K0F and 2A4J. The bound peptides open the protein binding sites, which enables targeting by other binders. In the case of 2K0F, including 160 models, we have chosen those 31 models giving the better superposition of the binding zone into the X-ray structure 1CLL. The residues 4-12 of 19-mer smMLCK peptide bound in 2K0F was considered to define the binding pocket. The residues 2-10 of P17-XPC peptide were used to define the binding site of C-HsCen2. Thus, for all selected protein structures of C-CaM and C-HsCen2, the pocket region involved the residues 88-142 and 112-166, respectively.

Using the on-line tool Fpocket [49] we calculated the volume and the local hydrophobic density of the binding pockets. The on-line tool PCE "Protein Continuum Electrostatics" [59] was used to calculate the pKa values of the titratable groups as well as the 3D electrostatic potential distribution of the C-terminal domains on the X-ray CaM (code 1CLL) and HsCen2 (code 2GGM) structures including the Ca^2+ ^atoms and taking dielectric constants of the solute and solvent as 11 and 80, respectively.

### Molecular docking

Figure 4 represents the entire workflow of the docking-scoring procedure. For all selected protein structures, the binding sites were prepared uniformly as input for docking experiments using the Dock Prep tool of Chimera [60]. Water molecules were removed from the protein binding sites and hydrogen atoms were added. The molecular surface of the receptor structures was computed using the program DMS [61] with a probe radius of 1.4 Å. For docking of 1-naphthyl terphenyl we used the program DOCK6.0 [45] accomplishing a sphere-matching algorithm via an "anchor-first" algorithm to fit ligand atoms to spheres representing a negative image of the receptor binding site. For ligand rotatable bonds we applied our optimized parameters [62] to better handle the ligand flexibility. The spheres were generated using the program SPHGEN [63]. We selected the set of spheres representing the binding site within 4 Å around the reference ligand, the bound peptides smMLCK and P17-XPC for C-CaM and C-HsCen2, respectively. The 3D structure of 1-naphthyl terphenyl was generated using the in-house program DG-AMMOS [64]. During the docking run, a maximum of 1000 orientations have been generated for each anchor and the DOCK grid energy score including electrostatic and van der Waals interactions was employed. The top 20 scored poses were retained for further consideration. In order to validate the docking performance of DOCK6.0 we performed self-docking test with trifluoperazine on the X-ray PDB structure of the CaM-trifluoperazine complex (code 1CTR, 1:1 complex) following the same protocol. Three of the top 20 scored poses showed RMSD with the bioactive trifluoperazine conformation of 1.5 - 2 Å which can be considered as good results keeping in mind the large binding pocket of CaM.

To evaluate the docking of 1-naphthyl terphenyl into CaM and HsCen2 we calculated the RMSD values between the docking poses and the bound peptides for each retained pose. The RMSD values were computed on the pharmacophoric points of 1-naphthyl terphenyl (see Figure 1) as follows: for CaM: the middle point between the atoms CD2 and CE2 of W4 (*i *residue) corresponding to the point 1, the CA atom of W4 corresponding to the point 1', and the atom CA of T7 (*i + 3 *residue) corresponding to the point 2; for HsCen2: the middle point between the atoms CD2 and CE2 of W2 corresponding to the point 1 (*i *residue), the atom CA of L5 (*i + 3 *residue) corresponding to the point 2, and the atom CA of L9 (*i + 7 *residue) corresponding to the point 3.

### Post-docking refinement and re-scoring

We used the open source program AMMOS recently developed by our group [52] for pose refinement on the best NMR and X-ray protein structures. We employed an energy minimization to refine all poses retained after DOCK6.0 docking on the selected protein receptor conformations allowing flexible ligand and flexible side chains of the receptor residues inside of a sphere with radius 6 Å around the ligand.

Next, we performed re-scoring on the AMMOS minimized docking poses with the Generalized Born/solvent accessible surface area (GBSA) method estimating the electrostatic/nonpolar contribution to solvation by employing the Hawkins GBSA method available in DOCK6.0. The Hawkins GBSA score is an implementation of the Molecular Mechanics Generalized Born Surface Area (MM-GBSA) method originally described by [65]. The Ca^2+ ^ions were included in the GBSA computations and the charges of titratable protein groups were assigned corresponding to the performed pKa calculations. The nonbonded van der Waals and electrostatic interaction terms were taken in the final GBSA scoring.

In addition, we performed re-scoring on the AMMOS minimized docking poses by using the program X-Score [54] developed for binding affinity estimation [66]. The X-Score empirical scoring functions implemented in X-Score, HSScore, HPScore and HMScore, include terms for: van der Waals interactions, hydrogen bonds, hydrophobic effects, a torsional entropy penalty and a regression constant. They differ in the manner of estimation of the hydrophobic effects. We used the averaged score of the three X-Score functions.

All structure figures were generated with PYMOL software [67].

## Authors' contributions

MAM, SM and CTC designed the project. AI and AB performed docking simulations. All authors participated in the analysis and manuscript writing. All authors read and approved the final manuscript.
